# Vivisection and Actual Facts

**Published:** 1914-06

**Authors:** 


**A Monthly Review of Medicine and Surgery**

EDITOR AND PUBLISHER DR. A. L. BENEDICT, 228 Summer St., corner of Elmwood Ave., Buffalo, (Address for all communications. Please make personal and telephone calls before 1 P. M.)

Third, in age, on the western continent. Independent, but supports professional organization. News limited mainly to Western N. Y. and Penn.

Subscription $2.00 a year; $3.00 for two years in advance. Changes and errors in address or failure or duplication of delivery, should be reported immediately.

Yearly Volume 69 JUNE, 1914 No. 11

### Vivisection and Actual Facts

It occurs to us that a good deal of the strength of the antivivisection movement depends on the lack of actual statistics and definite information, and the impression which antivivisectionists are able to create in the minds of many people, that the medical profession is not willing to come out in the open. A good deal of the lack of harmony is due to the fact that the antis deal fortissimo with physiologic experiments without anaesthesia and the defenders of animal experimentation fortissimo with serums and antigens. Some of the latter arguments have impressed us as implying a much more direct connection with animal experimentation, in vivo, than is actually the case, and much more brilliant results in therapeutics than experience warrants. Frank admission is one of the strongest points in logic and an argument that produces one impression on one's antagonist and is intended to produce another on one's supporters, is essentially weak.

For instance, it might as well be admitted that vivisection, up to the period of the discovery and use of anaesthetics, was, with trivial exceptions, extremely painful. The usual descriptions of curare in medical text books warrant a similar conclusion in regard to its experimental use. Such descriptions do not, so far as we can interpret them, suggest any superiority of curare over ordinary anaesthetics, even from the nonsentimental physiologic standpoint. They suggest further the sceptic inquiry as to whether curare has a special affinity for motor nerves, both from the stand point of a prior probability and as implied in the question as to how human beings subjected to the action of curare, can inform us as to this highly specialized action. An investigation in this particular might perhaps eliminate one of the best "talking points" of anti-vivisectionists. Even the practical economic difficulties in the way of obtaining curare and its apparent lack of superiority over anaesthetics, suggest further that actual statistics as to its use might have the same effect, at least for present purposes.

It is a well known fact that pain, however caused, interferes to a marked degree with various reflexes. This is demonstrated even by the simplest clinical observation. For example, in regard to absorptive motor, and secretory phenomena of the alimentary canal, pain produces abnormalities of the most conspicuous form. There are whole fields of physiologic and pharmacologic research in which, so far as can be judged from such analogy, pain is far more important as an interfering factor than anaesthesia. The old painful vivisections undoubtedly revealed physiologic facts of great fundamental importance. But, from the modern standpoint, they are crude. It is scarcely conceivable that their repetition would either elucidate more recondite problems or that any form of animal experimentation would serve the purpose of clinical examinations and analyses in justifying conclusions as to strictly human physiology.

These facts are not comprehended by the laity. Indeed, even the laity who are disposed to favor animal experimentation in the interests of humanity—including ultimate welfare of animals—are often imbued with the entirely false notion that the greater the pain, the greater the value of an experiment. On the contrary, as a general rule, a painful experiment in a stupid experiment. There are exceptions to this rule but, so far as we know, they have never been systematically formulated.

A large proportion of modern animal experimentation involves captivity, peculiarities of diet, artificially produced disease, simple procedures such as hypodermic injection, venesection, observation of excretions, taking of temperatures, etc., which are either entirely lacking in pain or involve only such trivial suffering as clinical observation and therapeutics of human patients involve. Rarely do they involve so much discomfort as are entailed in the keeping of live stock for food or even for wool, milk, eggs, feathers, etc.

As stated, some of the defenders of vivisection have given rather exaggerated impressions of the results of animal experiment. Speaking merely as a practitioner, this is unfortunate. It is highly complimentary but decidedly disconcerting to encounter a patient who has the impression that such study has yielded a knowledge of normal and perverted function such as to obviate the necessity of immediate clinical research or who expects that there is an appropriate serum capable of effecting a prompt cure in a large variety of affections. Even in the medical profession, the same exaggerated ideas are occasionally encountered. As a matter of fact, all the biologic products of marked efficacy against specific infections can be counted on the fingers, perhaps even of one hand. By no means all of these have any direct connection with animal experiment. It is also worth while for various reasons, to give due credit to morbid anatomy, clinical chemistry, the limitation of symptoms to lesions by observation of human symptomatology, noting post mortem changes and the results of operation. We have seen it stated that typhoid fever is one of the diseases whose control depends upon animal experimentation. Now, while such experimentation has yielded some very interesting results, they are mainly negative, confirming the previous conception of typhoid as essentially a human disease, excepting for certain results under highly artificial conditions, scarcely conforming to the strict definition of disease, and it is only in a far-fetched sense that animal experimentation has anything to do with either the sanitary or vaccine control of typhoid.

A thorough study of animal experimentation, as actually carried on, would undoubtedly be of great value to medical science. Such a study should include a careful scrutiny of actual results, scientific and practical as well as statistics of interest mainly from the standpoint of humane societies. For example, a historic review of the pre-anaesthetic period would determine what fundamental facts in physiology and pathology, had been determined by experiment, how far these determinations corroborated accepted theories or facts otherwise demonstrated and how far they corrected misconceptions. The proper value of animal experimentation, human symptomatology and checking of results by post mortem and by operation, clinical diagnostic methods, etc., should be accurately compared. The results of pharmacologic and toxicologic observations, of diagnostic reactions, th utilization of animals for obtaining biologic products, for acquiring surgical dexterity and judging probable results of operations, for demonstrating accepted physiologic facts to students, for observations of dietetic effects and those of other artificial conditions, etc., would include one range of study.

From the standpoint of humane societies, a somewhat different line would be followed. Thus, we should distinguish experiments which involved merely observation terminated by death, utilization for the preparation of biologic material, for diagnostic or therapeutic purposes, literal vivisections under full anaesthesia, terminated by death without recovery of consciousness, experimental operation under anaesthesia with subsequent observation, artificial production of disease; as by inoculation, painful experiments, with frank statement of scientific and practical value of results, use of curare, etc.

We feel confident that a systematic, statistic study of this nature would afford the profession a strong case, that it would clarify impressions in the profession itself, that it would altogether destroy certain arguments of the antis, that it would limit controversy, among all reasonable persons, to a very limited field, and that it would do away with the annual menace of drastic legislation, largely initiated on account of misconceptions and fostered by the apparent attitude of the profession in concealing actual conditions.

